# Recurrent Glioma With Lineage Conversion From Oligodendroglioma to Astrocytoma in Two Cases

**DOI:** 10.3389/fonc.2019.00828

**Published:** 2019-08-27

**Authors:** Jo-Heon Kim, Woo-Youl Jang, Tae-Young Jung, Shin Jung, Kyung-Keun Kim, Hyung-Seok Kim, Eun-Hee Kim, Min-Cheol Lee, Kyung-Sub Moon, Kyung-Hwa Lee

**Affiliations:** ^1^Department of Pathology, Chonnam National University Research Institute of Medical Science, Chonnam National University Hwasun Hospital and Medical School, Hwasun, South Korea; ^2^Department of Neurosurgery, Chonnam National University Research Institute of Medical Science, Chonnam National University Hwasun Hospital and Medical School, Hwasun, South Korea; ^3^Department of Pharmacology, Chonnam National University Medical School, Gwangju, South Korea; ^4^Department of Forensic Medicine, Chonnam National University Medical School, Gwangju, South Korea

**Keywords:** cell lineage, clonal evolution, genetic heterogeneity, mixed oligoastrocytoma, 1p/19q-codeletion, recurrent glioma

## Abstract

Following the introduction of the molecular classification of gliomas by the WHO in 2016, molecularly-proven lineage conversion during glioma recurrence has never been reported. The reported two cases were initially diagnosed as oligodendroglioma with 1p/19q-codeletion and mutation of *isocitrate dehydrogenase 1 (IDH1)*-R132H. The recurrent tumors showed loss of alpha-thalassemia/mental retardation X-linked (ATRX) expression, strong P53 positivity, and 1p/19q-nondeletion. Next generation sequencing analysis performed on the first case confirmed the transition of molecular traits from oligodendroglioma to astrocytoma. An *IDH* mutation of R132H was preserved in the episodes of recurrence, but *ATRX* and *TP53* mutations were newly acquired and *TERT* promoter mutation C228T was lost at the most recent recurrence. The issue in question for the presented cases is whether the original tumors were pure oligodendrogliomas that then transdifferentiated into astrocytomas, or whether the original tumor was an oligoastrocytoma having oligodendroglioma cells that outnumbered the astrocytoma cells and where the astrocytoma cells becoming more dominant over the episodes of recurrence. With the recognition of the possibility of lineage conversion, our study suggests that molecular examination should be performed to adjust therapeutic strategies in recurrent gliomas. Indeed, our observation of lineage conversion in glioma recurrence calls into question the current distinction drawn between oligodendroglioma, astrocytoma and oligoastrocytoma, rather than simply bidding “farewell to oligoastrocytoma.”

## Background

Following the introduction of the WHO central nervous system tumor classification in 2016, the presence of an oligodendroglioma component must be proved by 1p/19q codeletion, and diagnosis of an astrocytoma population must be supported by *Alpha-thalassemia/mental retardation X-linked (ATRX)* loss and/or a *TP53* mutation, in addition to trunk *isocitrate dehydrogenase (IDH)* 1/2 mutations (1). Before the introduction of the narrow definition of oligodendroglioma based on the presence of an *IDH* mutation and 1p/19q codeletion, the diagnosis of oligoastrocytoma was based on somewhat subjective criteria. Histological features from hematoxylin and eosin staining were loosely interpreted to decide a “conspicuous mixture” of oligodendroglioma and astrocytoma populations (2). According to the new classification scheme, “oligoastrocytoma, NOS” (not otherwise specified) is reserved for unclassified gliomas having no molecular information. Oligoastrocytoma with a dual genotype could be considered as a “true oligoastrocytoma” rather than oligoastrocytoma, NOS (3–5). Although the reported cases displayed components of both oligodendroglioma and astrocytoma that were proven by molecular examination, the current classification does not accept the dual-genotype oligoastrocytoma as a distinct entity.

In glioblastoma (GM), genetic heterogeneity in multifocal tumors or genetic evolution along with treatment has been well-studied (6–11). However, a small number of studies have focused on collective molecular signatures rather than on lineage clarification in lower-grade gliomas (LGG) (12–14). This molecular information of a tumor tends to be derived from a group of tumor cells and does not fully reflect the status of small populations of tumor cells, nor relate to the single-cell level. Based on the strict diagnostic criteria for LGG based on the differentiation lineages of oligodendroglioma or astrocytoma, genetic heterogeneity or evolutionary changes over episodes of recurrence, in molecular terms, have rarely been elucidated in lower-grade glioma (15–17). Although lineage conversion in recurrent LGG has been reported in treatment-naïve multicentric tumors (17) or only with histological classification (10), molecularly-proven lineage conversion along with the treatment has never been reported. Herein, we report two cases of recurrent glioma that began as oligodendroglioma and converted to astrocytoma over episodes of recurrence.

## Materials and Methods

Representative tissue blocks were immunostained with specific antibodies against IDH1-R132H (dilution 1:200, product no. DIA-H09; Dianova, Hamburg, Germany), P53 (dilution 1:600, product no. M7001; DAKO, Glostrup, Denmark), ATRX (1:200, product no. HPA001906; Sigma, St. Louis, MO, USA). 3-μm sections were cut from formalin-fixed, paraffin-embedded (FFPE) blocks and subjected to IHC staining using an automated immunostainer (Bond-maX DC2002; Leica Biosystems, Bannockburn, IL, USA). Programmed heat-induced epitope retrieval was carried out using bond epitope retrieval solution 1 (containing citrate buffer at pH 6.0) or 2 (containing Tris EDTA, pH 9.0) for 15 min.

Dual-color FISH analysis of chromosomal loci 1p36 and 19q13 was performed using commercially available probes (Abbott/Vysis, Chicago, IL, USA) according to the manufacturer's instructions, as previously described (18). Briefly, 1-μm-thick unstained sections of representative paraffin blocks were prepared. Target probes carrying an orange fluorophore were directed at either 1p36 or 19q13, and reference probes with a green fluorophore were directed to 1q25 or 19p13. Fluorescent signals were counted using a Nikon Eclipse 80i microscope with appropriate filters (Nikon, Tokyo, Japan) and the Isis imaging program (MetaSystems GmbH, Altlussheim, Germany). Following overnight probe incubation, the slides were counter-stained with 4′,6-diamidino-2-phenylindole (DAPI). One hundred non-overlapping tumor nuclei were enumerated and analyzed for loss of the orange target signal. For each case, the ratio of the test probe to the reference probe was ≤0.8, and the absence of one orange target signal, with retention of two green reference signals within at least 25% of the counted nuclei, defined deletion of the analyzed locus.

## Next-Generation Sequencing Analysis

Three tissue samples of the first case were obtained. DNA was extracted from macrodissected tissue on 10-μm-thick unstained FFPE sections and purified using the QIAamp DNA FFPE Tissue Kit (QIAGEN GmbH, Hilden, Germany) according to the manufacturer's instructions. A targeted panel was used to capture the target region of 83 genes, which included all coding exons of the gene for detection of single-nucleotide variants (SNVs), insertions/deletions (INDELs), and copy number variants (CNVs) (Supplementary Data Sheet 1). Genomic DNA (200~500 ng) was prepared to construct libraries using the SureSelect targeted panel (Agilent, Santa Clara, CA, USA) according to the manufacturer's protocol. Briefly, the qualified genomic DNA sample was randomly fragmented by Covaris sonication (Woburn, MA, USA) followed by adapter ligation, purification, hybridization, and PCR. The captured libraries were subjected to the Agilent 2100 Bioanalyzer to estimate the quality and loaded onto the Illumina HiSeq 2500 instrument (San Diego, CA, USA) of Theragen-Etex Bio Institute (Suwon, Korea) according to the manufacturer's recommendations. Raw image files were processed by HCS1.4.8 for base-calling with default parameters, and the sequences of each sample were generated as 101-bp paired-end reads. At the NGS data pre-processing step, the sequence reads were aligned to the human genome (hg19) using BWA-MEM (19). For analysis-ready BAM file generation, the overall pre-processing procedure, including duplication removal, local realignment, and recalibration, was performed according to GATK Best Practices recommended by the Broad Institute (20). At the variant discovery step, SNVs and INDELs were analyzed using three open-source callers [UnifiedGenotyper (21), Freq (22), SNVer (23), in addition to the in-house callers of Samsung SDS's (Sungnam-Si, South Korea)]. CNVs and translocations were discovered using in-house callers developed by Samsung SDS. SNVs and INDELs were detected with ensemble methods integrating three open source callers with the in-house caller. SNVs and INDELs were filtered using germline mutations and false-positive filters. SNVs with a variant allele frequency (VAF) ≥3% and INDELs ≥10% were selected as the final result. CNVs were analyzed according to the depth of coverage of each target region between the tumor and using pre-processed (normalized) data. To calculate the absolute copy number, the tumor purity and ploidy were estimated by a statistical model using log_2_ ratio and SNV VAF values. Cut-off values of CN ≥ 7 and CN = 0 were used for amplification and homo-deletion, respectively. Institutional Review Board of our hospital approved this study (CNUHH-2018-015), and written informed consent was obtained from patients.

## Case Presentation

### CASE 1

#### Clinical History

**Figure 1:**
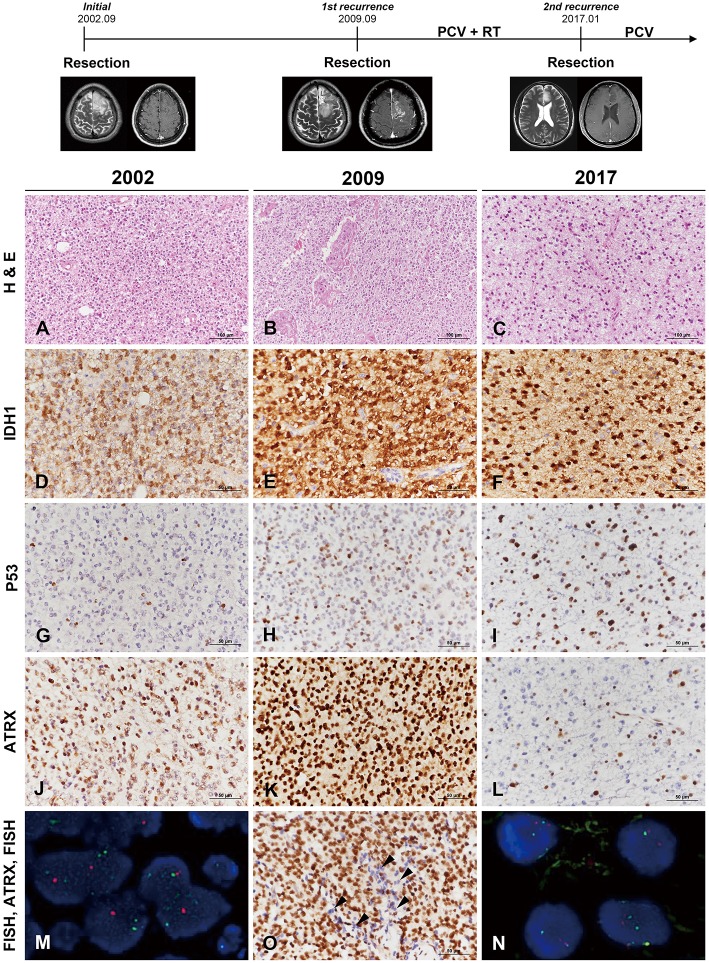
Clinicoradiological and pathological findings in case 1. The clinical progression of case 1 is summarized in uppermost. In 2002, the lesion was composed of round monotonous tumor cells with perinuclear halos **(A)** and showed increased cellularity with microvascular proliferation in 2009 **(B)**. The most recent tumor, in 2017, displayed relatively low cellularity but increased cytological atypia **(C)** (**A–C**, hematoxylin and eosin staining; original magnification, × 200). The tumor cells showed well-preserved IDH1-R132H positivity at each recurrence **(D–F)**, and P53 staining intensity became stronger with further recurrences **(G–I)**. In comparison, ATRX expression was well-maintained according to two earlier biopsies **(J,K)** but was lost in the recent recurrence episode **(L)**. Interestingly, the specimen in 2009 demonstrated strong ATRX expression but also contained tumor cells showing loss of ATRX expression in small areas (**O**, arrowheads) (**D–L,O**, IHC results; original magnification, × 400). Chromosome 1 FISH performed on the 2002 specimen demonstrated loss of 1p (red) in the tumor cells **(M)**. Chromosome 19 FISH performed on the 2017 specimen demonstrated two copies of 19q (red) and 19p (green) in the tumor cells **(N)** (**M,N**, FISH; original magnification, × 1,000). ^*^ATRX, alpha-thalassemia/mental retardation X-linked; FISH, fluorescence *in situ* hybridization; IDH, isocitrate dehydrogenase; IHC, immunohistochemistry.

Clinical course and radiological features are summarized in Figure 1 (uppermost). A 29-year-old man was transferred to our hospital complaining of generalized tonic-clonic seizure. Initial MR imaging revealed a 7 × 4 × 5-cm ill-defined mass with subtle enhancement in the left frontal area. Gross total resection was carried out, and the diagnosis was of WHO grade II oligodendroglioma. A definitive single recurred mass was found in the same left frontal area on MR imaging without evidence of multifocality 7 years after the initial diagnosis. Re-operation was performed, and the lesion was diagnosed as anaplastic oligodendroglioma (AO). The patient received three cycles of PCV (procarbazine, CCNU [Lomustine], and vincristine) chemotherapy followed by radiation treatment. There was recurrence of the mass as a single lesion in the left frontal area again 7.5 years after the second operation. Surgical resection was retried to confirm the pathology and the lesion was diagnosed as anaplastic astrocytoma (AA). PCV chemotherapy was retried at the out-patient department.

#### Pathological Findings

The original lesion at the age of 29 years was a diffusely infiltrating tumor composed of round, uniform cells with perinuclear halos (Figure 1A). The tumor was moderately cellular and had no anaplastic features, including necrosis, microvascular proliferation, or brisk mitotic activity. The tumor was diagnosed as oligodendroglioma solely based on the histological examination. The first recurrent tumor 7 years after the initial surgery was highly cellular and exhibited nuclear atypia. Intratumoral microvessels were proliferative, with increased endothelial layers (Figure 1B). The pathological findings were consistent with AO. Seven-and-a-half years after the second operation, the patient again showed tumor recurrence. The second recurrent tumor, obtained from the third surgery, was composed of oval-to-round nuclei with mild nuclear atypia (Figure 1C). The tumor cells of initial and recurrent were positive for IDH1-R132H (Figures 1D–F). P53 staining intensity was negative in most of the initial tumor cells and became stronger with further recurrences (Figures 1G–I). ATRX preservation was observed in the initial and first recurrent tumor (Figures 1J,K). The second recurrent tumor showed the loss of ATRX (Figure 1L). Interestingly, a small number of tumor cells of the first recurrent mass demonstrated ATRX loss and were intermingled with ARTX-preserving cells (Figure 1O). 1p/19q FISH examination performed on the first two tumors revealed 1p/19q codeletion (Figure 1M), while the second recurrent tumor did not (Figure 1N). Contrary to the two previous tumors, the third tumor presented with the histological and molecular features of AA.

#### Mutation Analysis by Next-Generation Sequencing

**Figure 2:**
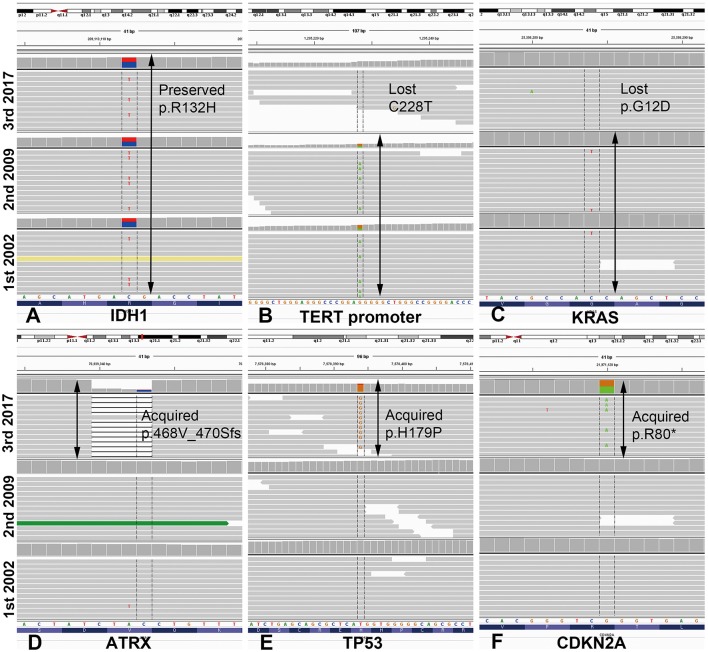
Illustration of mutations detected by next-generation sequencing analysis in case 1, visualized in Integrative Genome Viewer. *IDH1* mutation R132H was preserved at the same loci as that of the original tumor in the most recent recurrence **(A)**. *TERT* promoter mutation C228T and *KRAS* mutation G12D were maintained from the original to the recurrent anaplastic oligodendroglioma, but were lost in the recurrent astrocytoma **(B,C)**. By contrast, mutations of *ATRX, TP53*, and *CDKN2A* were newly acquired at the most recent astrocytoma recurrence. Arrows indicate the retention of the same mutations at each recurrence **(D–F)**. ^*^ATRX, alpha-thalassemia/mental retardation X-linked; IDH, isocitrate dehydrogenase.

We performed mutation analysis on three sequential specimens of the first patient using the SureSelect gene panel, which includes the most powerful genes for identifying the histological type of a glioma (e.g., *IDH1/2, ATRX, TP53, TERT*, and additional 78 genes). The mutations detected by NGS are summarized in Figure 2 and Supplementary Data Sheet 2. To exclude accidental mix-up of samples, Y-chromosome specific short tandem repeat (Y-STR) analysis was performed on three sequential samples of the first patient. The analysis result showed that all FFPE samples belonged to the same patient (Supplementary Data Sheet 3). Of the mutations listed, those that had been preserved over the episodes of recurrence were *IDH1-R132H, FBXW7, NOTCH1, BRCA1*, and *GNAS*. Mutations of *ATRX* and *TP53*, which are near-exclusive markers of astrocytoma, were evident at the second recurrence from AO to astrocytoma. *CDKN2A* mutation was also seen at the second recurrence. By contrast, the *TERT* promoter C228T and *KRAS* G12D mutations were lost at the second recurrence (vcf files in Supplementary Files 4–6 inside Supplementary Data Sheet 4). Despite scrupulous review of the NGS data, none of the previous oligodendroglioma or AO showed reads with variant alleles harboring *ATRX* or *TP53* genes (total VAF = 0.000%, both).

### CASE 2

#### Clinical History

**Figure 3:**
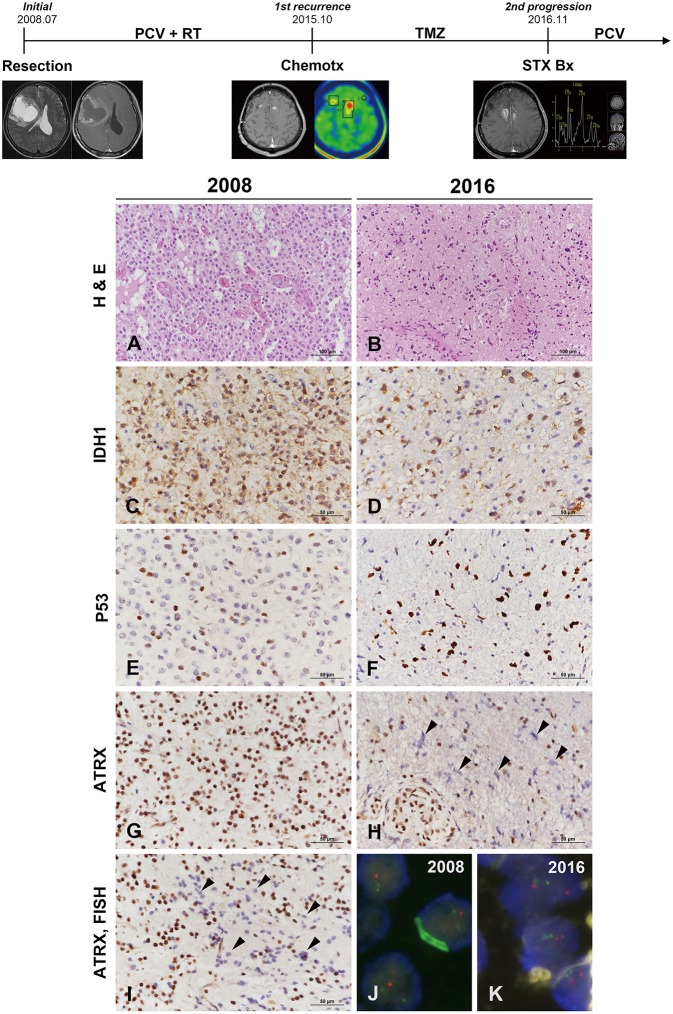
Clinicoradiological and pathological findings in case 2. The clinical progression of case 2 is summarized in uppermost. The original lesion displayed microvascular proliferation among monotonous tumor cells with perinuclear halos in 2008 **(A)** and then converted to a tumor with marked cytological atypia in 2016 **(B)** (**A,B**, hematoxylin and eosin stain; original magnification, × 200). The tumor cells showed IDH1-R132H positivity by IHC at both occasions **(C,D)**. P53 immunoreactivity converted from weak heterogeneous positivity of the wild type in 2008 to strong positivity of the mutant type at recurrence **(E,F)**. ATRX expression was strongly positive in the original tumor **(G)** but was lost at recurrence **(H)**. Similar to case 1, the specimen in 2009 demonstrated ATRX-negative cells that intermingled with the cells showing strong ATRX expression (**I**, arrowheads) (**C–H,I**, IHC results; original magnification, × 400). Chromosome 1 FISH performed on the 2002 specimen demonstrated loss of 1p (red) in the tumor cells **(J)**. Chromosome 19 FISH performed on the 2016 specimen demonstrated two copies of 19q (red) and 19p (green) in the tumor cells **(K)** (**J,K**, FISH; original magnification, × 1,000). ^*^ATRX, alpha-thalassemia/mental retardation X-linked; FISH, fluorescence *in situ* hybridization; IDH, isocitrate dehydrogenase; IHC, immunohistochemistry.

Clinical course and radiological features are summarized in Figure 3 (uppermost). A 49-year-old male patient was admitted to our hospital with left hemiparesis (motor grade IV/IV). MR imaging showed a 7 × 7 × 6-cm cystic mass with heterogeneous enhancement in the right frontal area. Subtotal tumor removal via craniotomy was performed and the mass was diagnosed as AO. The patient received three cycles of PCV chemotherapy followed by radiation treatment. New multiple small-enhancing nodules observed in the both superior frontal gyri, both cingulate gyri, corpus callosum and both basal ganglia on MR imaging were confirmed as metabolically active lesions by methionine-positron emission tomography scan 7 years after the initial diagnosis. Despite a sixth cycle of temozolomide adjuvant chemotherapy, the lesion was progressively enlarged with tumoral spectrum on MR spectroscopy. To confirm the pathology, stereotactic biopsy with the Leksell frame was performed, and the lesion was diagnosed as glioblastoma. PCV chemotherapy was retried, but the neurological status of the patient had gradually improved.

### Pathological Findings

The original mass from a 49-year-old male was a highly cellular tumor composed of monotonous cells with perinuclear halos. The tumor showed geographic necrosis and microvascular proliferation in compact tumoral areas (Figure 3A). The tumor was diagnosed as AO based on histological examination and 1p/19q codeletion by FISH examination. The small recurrent mass specimens obtained via stereotactic biopsy 7 years after the initial diagnosis showed sparse cellularity. The lesion was composed of highly pleomorphic tumor cells and microvascular proliferation was present (Figure 3B). IHC of origin & recurrent tumors showed positive for IDH1-R132H (Figures 3C,D). P53 staining of origin tumor was that of the wild type but the pleomorphic cells of recurrent tumor were strongly positive for P53 (Figures 3E,F). While the origin tumor cells showed preservation of ATRX expression, the recurrent tumor displayed ATRX loss (Figures 3G,H). Strong P53 positivity, ATRX loss and 1p/19q-nondeletion together evidenced differentiation toward astrocytoma. Similar to the first recurrent tumor of case 1, a small number of tumor cells in the recurrent tumor exhibited ATRX loss among those with strong ATRX expression (Figure 3I). 1p/19q FISH examination performed on the initial tumors revealed 1p/19q codeletion (Figure 3J), while the recurrent tumor did not (Figure 3K). The recurrent tumor presented with the histological and molecular features of GM. The second case was not suitable for NGS analysis, because the DNA quantity could not be guaranteed due to the tiny tumor tissues derived from stereotactic biopsy.

## Discussion

The two cases of glioma presented in the current report began as oligodendroglioma with stereotypic characteristics of oligodendroglioma that included *IDH1* mutation, P53 negativity, and, most importantly, 1p/19q codeletion. However, the tumors converted from oligodendroglioma to astrocytoma with a long clinical period. Previously, studies analyzing on matched pairs of primary and recurrent oligodendrogliomas did not observe substitution of 1p/19q-nondeleted cells for 1p/19q-codeleted cells (15, 16). We devised two hypotheses to explain the lineage change seen in our cases.

The first hypothesis was that there was a basic *IDH* mutation accompanied by 1p/19q codeletion at tumor initiation, with *ATRX* loss and *TP53* mutation being newly acquired over the episodes of recurrence. If this hypothesis was proven, transdifferentiation with lineage conversion during tumoral evolution could be considered another facet of glioma recurrence, in addition to tumor progression toward a high-grade tumor. Increasing body of evidence has shed light on the genetic evolution in GM recently (6–11). Considering that multifocal or multicentric GMs are more prominent in genomic heterogeneity and contain various clonal genetic alterations than single tumor, genomic evolution may be presumed to occur simultaneously with the initial clonal divergence (6, 7). Genetic heterogeneity of GM was found to be higher in long-term recurrent tumors with multifocality (7). In LGG, clonal diversity or evolution related with multicentricity was recently investigated using NGS (17). Of the analyzed cases, included was one interesting case that was initially diagnosed as oligodendroglioma and then developed astrocytoma at a different site after 4 years without intervening chemoradiation. Compared with the case, the uniqueness of our cases is that the lineage conversion of the glioma happened at the same sites over recurrences with a long progressive period of 8, 15 years.

The second hypothesis was that the tumor cell population at the initial diagnosis was predominantly oligodendroglioma cells mixed with small amount of astrocytoma cells. Oligodendroglioma with 1p/19q codeletion has been known to show a better response to chemotherapy (24). Over the clinical course, the treatment-sensitive oligodendroglioma cells with 1p19q codeletion might have been perished by PCV chemotherapy and then outnumbered by overgrown astrocytoma cells. If proven, the second hypothesis would support that the pre-existing oligoastrocytoma may have been masked by predominant oligodendroglioma. In the present two cases, loss of ATRX expression was highly suspected based on IHC before the change of histological type (Figures 1O, 3I), although the number of tumor cells was very small. Unfortunately, the minor tumor components were difficult to show by means of molecular evaluation including the maximized depth of read by NGS analysis.

In the previously reported cases of dual-genotype oligoastrocytoma, the oligodendroglioma and astrocytoma components shared identical *IDH* mutations, of either *IDH1 R132H* (4, 5) or *IDH2* (3). Similar to the previously suggested hypothesis that an IDH mutation precedes tumorigenesis of the glioma (25), in the current cases trunk mutation of *IDH1-R132H* was preserved over the episodes of recurrence. According to the NGS panel analysis performed in our first case, there appeared to be a subset of genes whose mutations persisted, along with the *IDH1* mutation, which included *FBXW7, NOTCH1, BRCA1*, and *GNAS*. In comparison, *KRAS* and *TERT* promoter mutations disappeared with lineage conversion from oligodendroglioma to astrocytoma, and vice versa for *ATRX* and *TP53*. The genes acting in concert with the *IDH1* mutation provide support for sustained monoclonality despite lineage conversion; the latter group of genes which showed differences between oligodendroglioma and astrocytoma cells. While the dual-genotype oligoastrocytoma is supported by spatial heterogeneity, the present cases are supported by temporal heterogeneity and extremely slow mutational accumulation over 8, 15 years. The tumor evolution timescales of the current cases were much longer than those reported during follow-up by studies of matched pairs of recurrent oligodendroglioma (median of 28–44 months) (15, 16).

The present cases suggest that lineage conversion may occur in recurrent gliomas treated with chemoradiotheray over a long clinical period. It cannot be definitively concluded that the mechanism of lineage conversion involves disparate survival of tumor cells in heterogeneous populations, or complex transdifferentiation according to genetic evolution. Accumulation and analysis of more cases are needed to identify the precise mechanism. Nevertheless, our study highlights several clinical implications with respect to the diagnosis and treatment of oligodendroglioma. Compared with the slow progression of pure oligodendroglioma, the outcome of astrocytoma is significantly worse (4). With the recognition of the possibility of lineage conversion, molecular examination should be performed to adjust therapeutic strategies in recurrent gliomas. Indeed, our observation of lineage conversion in glioma recurrence calls into question the current distinction drawn between oligodendroglioma, astrocytoma and oligoastrocytoma, rather than simply bidding “farewell to oligoastrocytoma” (1, 24).

## Data Availability

All datasets generated for this study are included in the manuscript and/or the Supplementary Files.

## Ethics Statement

Institutional Review Board of our hospital approved this study (CNUHH-2018-015), and written informed consent was obtained from patients. Written informed consent was also obtained from the participant for the publication of this manuscript, including any potentially-identifying images or data.

## Author Contributions

K-HL and K-SM designed this study. K-HL, M-CL, and J-HK carried out the pathological studies. W-YJ, J-HK, K-HL, and K-SM drafted the manuscript. K-SM, W-YJ, and SJ carried out the acquisition and analysis of clinical data. K-HL, K-SM, SJ, K-KK, and T-YJ carried out the interpretation of clinical and experimental data. T-YJ, K-KK, M-CL, and SJ revised the manuscript for critical points. H-SK and E-HK carried out Y-STR analysis and interpreted the results. All authors read and approved the final manuscript.

### Conflict of Interest Statement

The authors declare that the research was conducted in the absence of any commercial or financial relationships that could be construed as a potential conflict of interest.

## Abbreviations

AO: anaplastic oligodendroglioma
ATRX: alpha-thalassemia/mental retardation X-linked
FISH: fluorescence *in-situ* hybridization
GM: glioblastoma
IDH: isocitrate dehydrogenase
IHC: immunohistochemistry
NGS: Next-generation sequencing
