# Supplementary material for: Recurrent Glioma With Lineage Conversion From Oligodendroglioma to Astrocytoma in Two Cases
Source: Front Oncol. 2019 Aug 27;9:828. doi: 10.3389/fonc.2019.00828 (PMC6719522; doi:10.3389/fonc.2019.00828)
Supplement: Supplementary file 1 [file Data_Sheet_1.docx]

Supplementary table S1. SureSelect panel list of 83 genes

| SNVs, INDELs, CNVs* | Rearrangement | Promoter |
| --- | --- | --- |
| ABL1 | ALK | TERT |
| AKT1 | RET |  |
| AKT2 | ROS1 |  |
| AKT3 |  |  |
| APC |  |  |
| ARID1A |  |  |
| ARID1B |  |  |
| ARID2 |  |  |
| ATM |  |  |
| ATRX |  |  |
| AURKA |  |  |
| AURKB |  |  |
| BCL2 |  |  |
| BRAF |  |  |
| BRCA1 |  |  |
| BRCA2 |  |  |
| CDH1 |  |  |
| CDK4 |  |  |
| CDK6 |  |  |
| CDKN2A |  |  |
| CNBB1 |  |  |
| CSF1R |  |  |
| DDR2 |  |  |
| EGFR |  |  |
| EPHB4 |  |  |
| ERBB2 |  |  |
| ERBB3 |  |  |
| ERBB4 |  |  |
| EWSR1 |  |  |
| EZH2 |  |  |
| FBXW7 |  |  |
| FGFR1 |  |  |
| FGFR2 |  |  |
| FGFR3 |  |  |
| FLT3 |  |  |
| GNA11 |  |  |
| GNAQ |  |  |
| GNAS |  |  |
| HNF1A |  |  |
| HRAS |  |  |
| IDH1 |  |  |
| IDH2 |  |  |
| IGF1R |  |  |
| ITK |  |  |
| JAK1 |  |  |
| JAK2 |  |  |
| JAK3 |  |  |
| KDR |  |  |
| KIT |  |  |
| KRAS |  |  |
| MDM2 |  |  |
| MET |  |  |
| MLH1 |  |  |
| MPL |  |  |
| MTOR |  |  |
| NF1 |  |  |
| NOTCH1 |  |  |
| NPM1 |  |  |
| NRAS |  |  |
| NTRK1 |  |  |
| PDGFRA |  |  |
| PDGFRB |  |  |
| PIK3CA |  |  |
| PIK3R1 |  |  |
| PTCH1 |  |  |
| PTCH2 |  |  |
| PTEN |  |  |
| PTPN11 |  |  |
| RB1 |  |  |
| SMAD4 |  |  |
| SMARCB1 |  |  |
| SMO |  |  |
| SRC |  |  |
| STK11 |  |  |
| SYK |  |  |
| TMPRSS2 |  |  |
| TOP1 |  |  |
| TP53 |  |  |
| VHL |  |  |

* CNVs, copy number variants; INDELs, insertions/deletions; SNVs, single-nucleotide variants;
