# Supplementary material for: Recurrent Glioma With Lineage Conversion From Oligodendroglioma to Astrocytoma in Two Cases
Source: Front Oncol. 2019 Aug 27;9:828. doi: 10.3389/fonc.2019.00828 (PMC6719522; doi:10.3389/fonc.2019.00828)
Supplement: Supplementary file 2 [file Data_Sheet_2.docx]

| Supplementary Table S2. Comparison of next gene sequencing analysis using SureSelect panel in the sequential specimens of the first case. | | | | | | | | | |  |
| --- | --- | --- | --- | --- | --- | --- | --- | --- | --- | --- |
| Gene | Position | Chromosome | Aa* | CDS* | 1st  OLG* | Total  VAF* (%) | 2nd  AO* | Total  VAF (%) | 3rd  AA* | Total  VAF (%) |
| IDH1 | 209113112 | 2 | p.Arg132His | c.395G>A | +* | 40.9 | + | 47.2 | + | 41.9 |
| FBXW7 | 153268226 | 4 | ― (splice site region) | ― (splice site region) | + | 64.4 | + | 64.5 | + | 69.6 |
| NOTCH1 | 139399155 | 9 | p.Arg1663Gln | c.4988G>A | + | 57.6 | + | 54.1 | + | 49.4 |
| BRCA1 | 41258531 | 17 | p.Leu52Phe | c.154C>T | + | 44.1 | + | 41.3 | + | 50 |
| GNAS | 57429663 | 20 | p.Asp448Ala | c.1343A>C | + | 12.7 | + | 12.4 | + | 22 |
| TERT promoter | 1295228 | 5 | ― (promoter) | ― (promoter) | + | 32.1 | + | 41.4 | – | – |
| KRAS | 25398284 | 12 | p.Gly12Asp | c.35G>A | + | 3.4 | + | 19.2 | – | – |
| TP53 | 7578394 | 17 | p.His179Pro | c.536A>C | –* | – | – | – | + | 87 |
| ATRX | 76939339 | X | p.468Val_470Serfs | c.1405_1407delGATG | – | – | – | – | + | 86.4 |
| CDKN2A | 21971120 | 9 | p.Arg80* | c.238C>T | – | – | – | – | + | 51.3 |

*+, present; –, absent; Aa, amino acid; AA, anaplastic astrocytoma; AO, anaplastic oligodendroglioma; CDS, coding sequence; OLG, oligodendroglioma; VAF, variant allele frequency
