# Supplementary material for: Recurrent Glioma With Lineage Conversion From Oligodendroglioma to Astrocytoma in Two Cases
Source: Front Oncol. 2019 Aug 27;9:828. doi: 10.3389/fonc.2019.00828 (PMC6719522; doi:10.3389/fonc.2019.00828)
Supplement: Supplementary file 3 [file Data_Sheet_3.pdf]

1. Material and Methods

Supplementary File 3

The PCR amplification after DNA extraction was performed according to PowerPlex Y23 user manual as previously described (26). PCR amplification was performed in individual 0.2mL thin-walled tubes on the GeneAmp® PCR System 9700 (Applied Biosystems-Life Technologies) at a total reaction volume of 25µL containing 5.0µL PowerPlex® Y23 5x Master Mix, 2.5µL PowerPlex® Y23 10x Primer Pair Mix, 7.5µL Amplification Grade Water, and 10µL normalized template DNA. Thermal cycling conditions consisted of enzyme activation at 96°C for 2 minutes, followed by 30 cycles of denaturation at 94°C for 10 seconds, annealing at 61°C for 1 minute, and extension at 72°C for 30 seconds. A final extension was carried out at 60°C for 20 minute with a final hold at 4°C.

Data were analyzed using GeneMapper® ID v 3.2.1 following the manufacturer's recommended analysis settings outlined in the user manual. All allelic ladders, positive amplification controls, and samples were reviewed for appropriate allele calls at each locus.

References

26. Gopinath S, Zhong C, Nguyen V, Ge J, Lagacé RE, Short ML, et al. Developmental validation of the Yfiler(®) Plus PCR Amplification Kit: An enhanced Y-STR multiplex for casework and database applications. Forensic Sci Int Genet. (2016) 24:164-175.

23 markers	2800M Control DNA
DYS576	18
DYS389 I	14
DYS448	19
DYS389 II	31
DYS19	14
DYS391	10
DYS481	22
DYS549	13
DYS533	12
DYS438	9
DYS437	14
DYS570	17
DYS635	21
DYS390	24
DYS439	12
DYS392	13
DYS643	10
DYS393	13
DYS458	17
DYS385	13, 16
DYS456	17
YGATAH4	11

2. Result

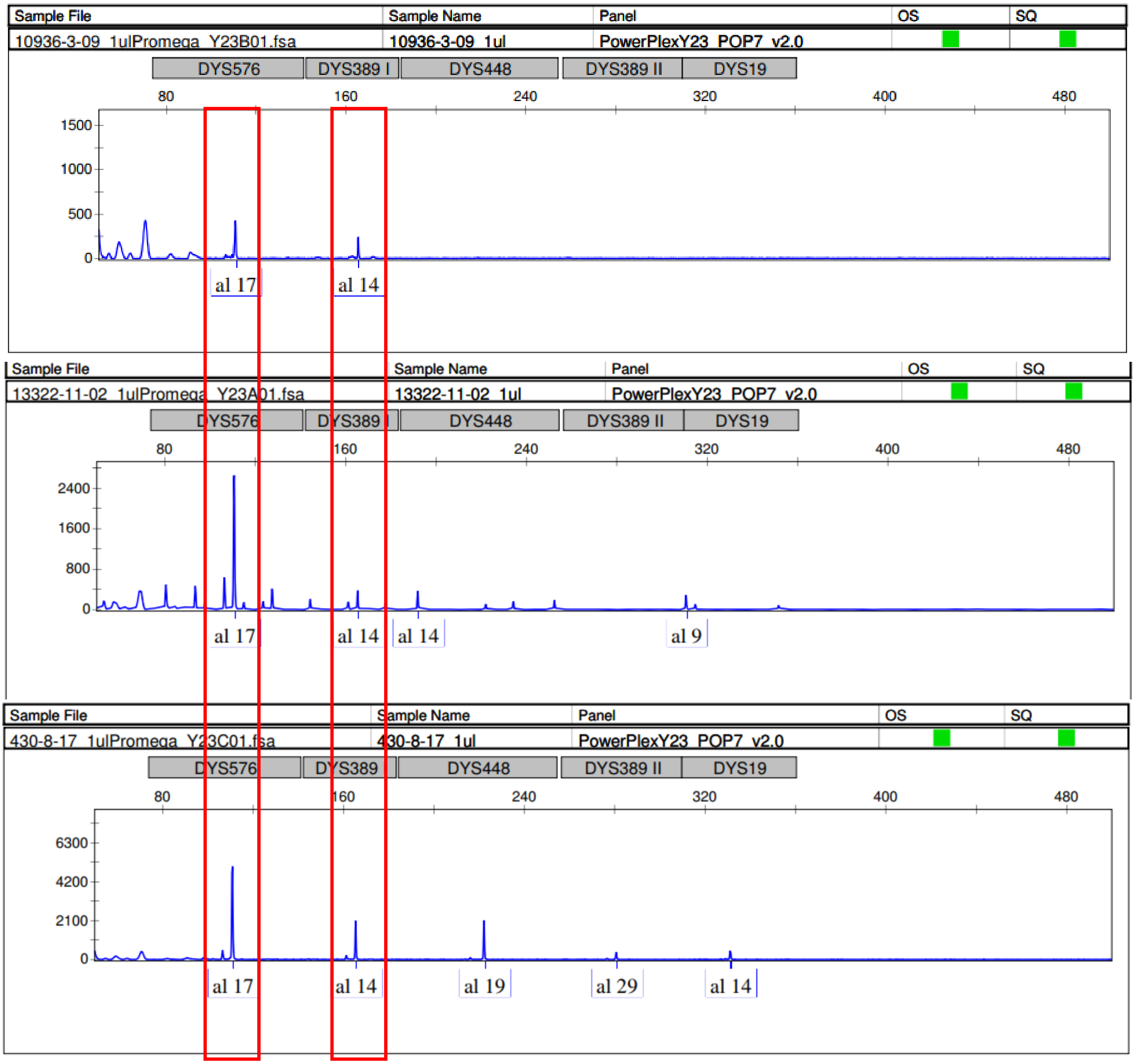

Ten markers (in red boxes) out of 23 were consistent between the samples.

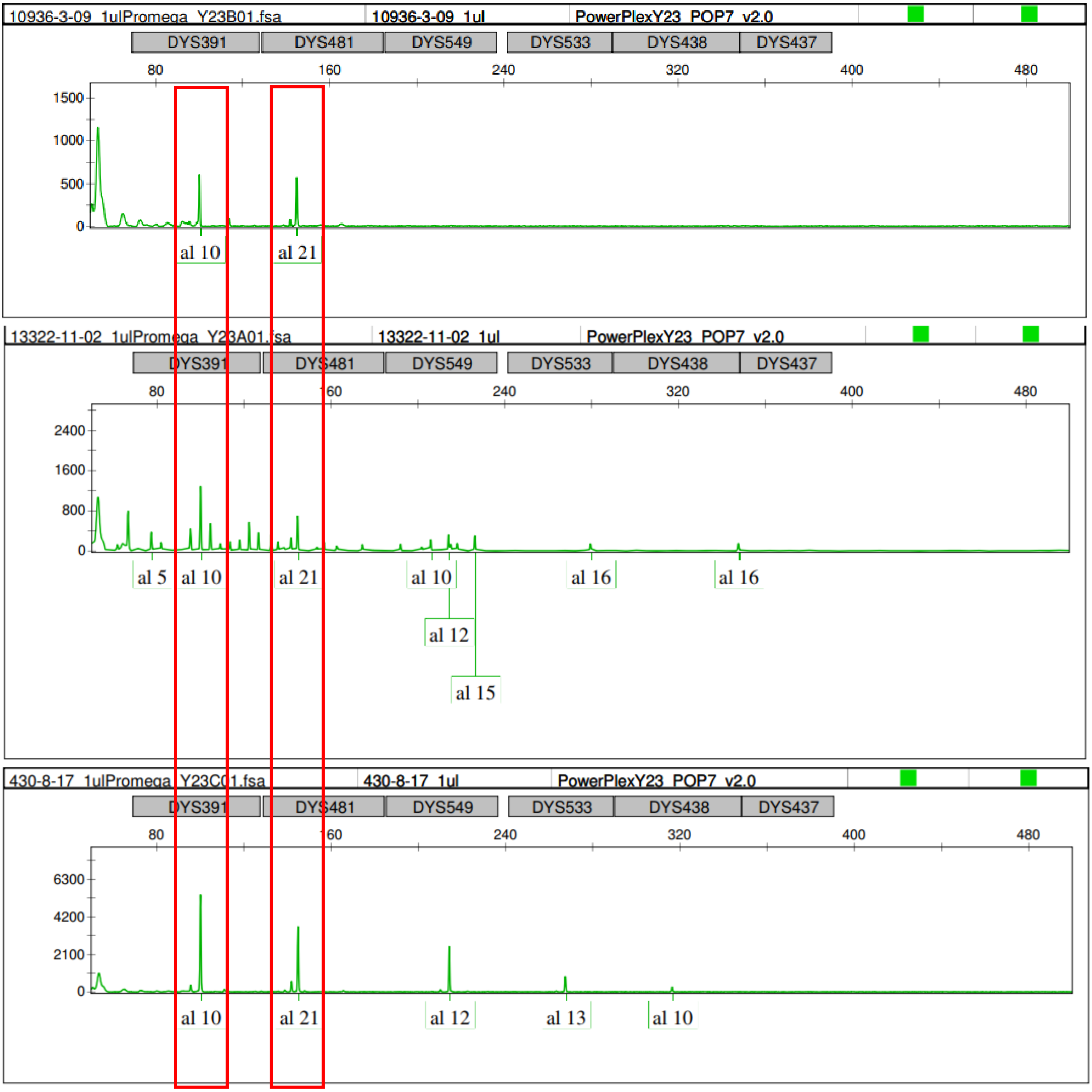
10936-3-09_1ulPromega_Y23B01.fsa
10936-3-09_1ul
PowerPlexY23_POP7_v2.0
DYS391
DYS481
DYS549
DYS533
DYS438
DYS437
80
160
240
320
400
480
1500
1000
500
0
al 10
al 21
13322-11-02_1ulPromega_Y23A01.fsa
13322-11-02_1ul
PowerPlexY23_POP7_v2.0
2400
1600
800
0
al 5
al 10
al 21
al 10
al 16
al 16
al 12
al 15
430-8-17_1ulPromega_Y23C01.fsa
430-8-17_1ul
PowerPlexY23_POP7_v2.0
6300
4200
2100
0
al 10
al 21
al 12
al 13
al 10

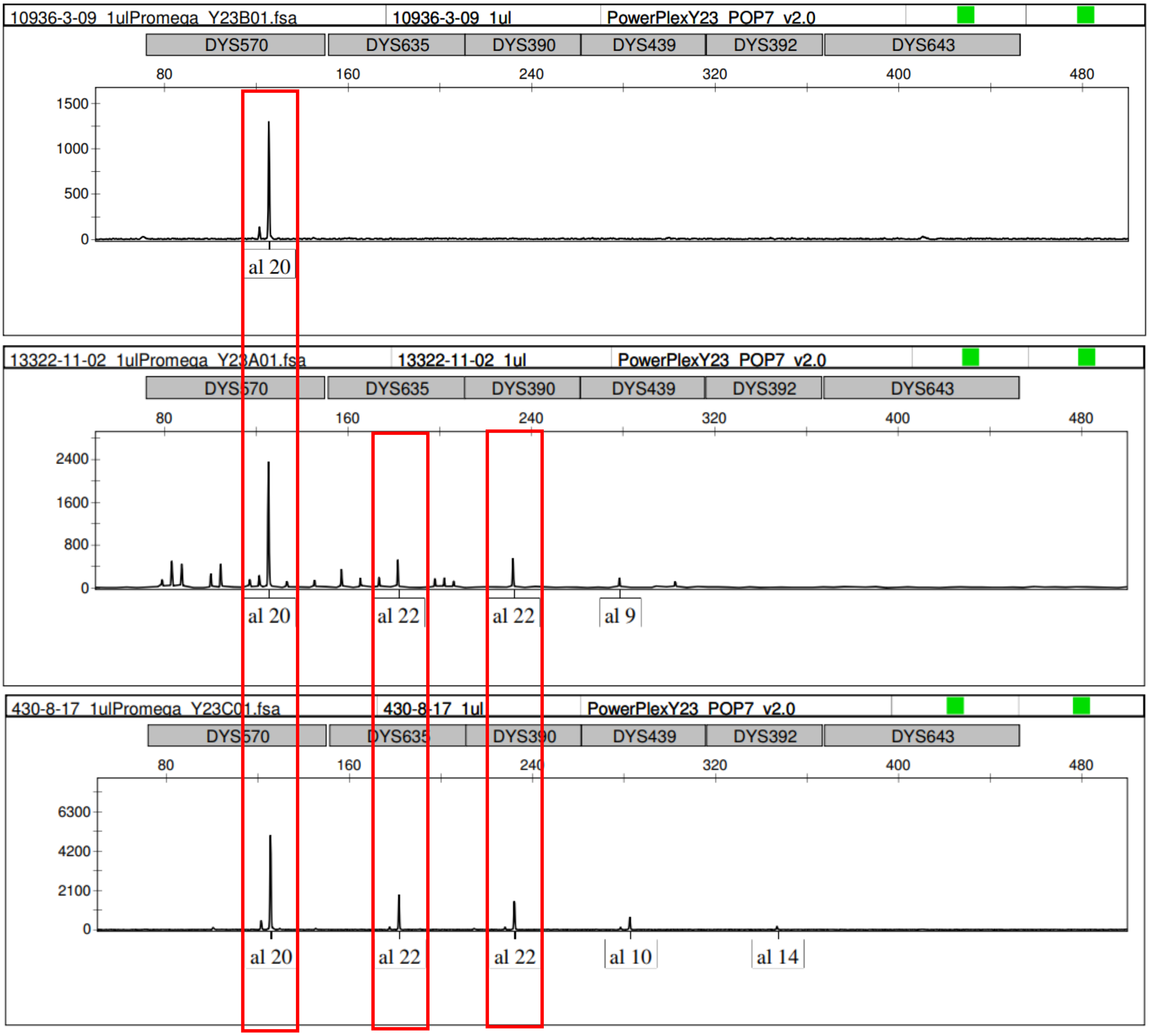
10936-3-09 1ulPromega Y23B01.fsa
10936-3-09 1ul
PowerPlexY23 POP7 v2.0
DYS570
DYS635
DYS390
DYS439
DYS392
DYS643
80
160
240
320
400
480
1500
1000
500
0
al 20
13322-11-02 1ulPromega Y23A01.fsa
13322-11-02 1ul
PowerPlexY23 POP7 v2.0
2400
1600
800
al 20
al 22
al 22
al 9
430-8-17 1ulPromega Y23C01.fsa
430-8-17 1ul
PowerPlexY23 POP7 v2.0
6300
4200
2100
al 20
al 22
al 22
al 10
al 14

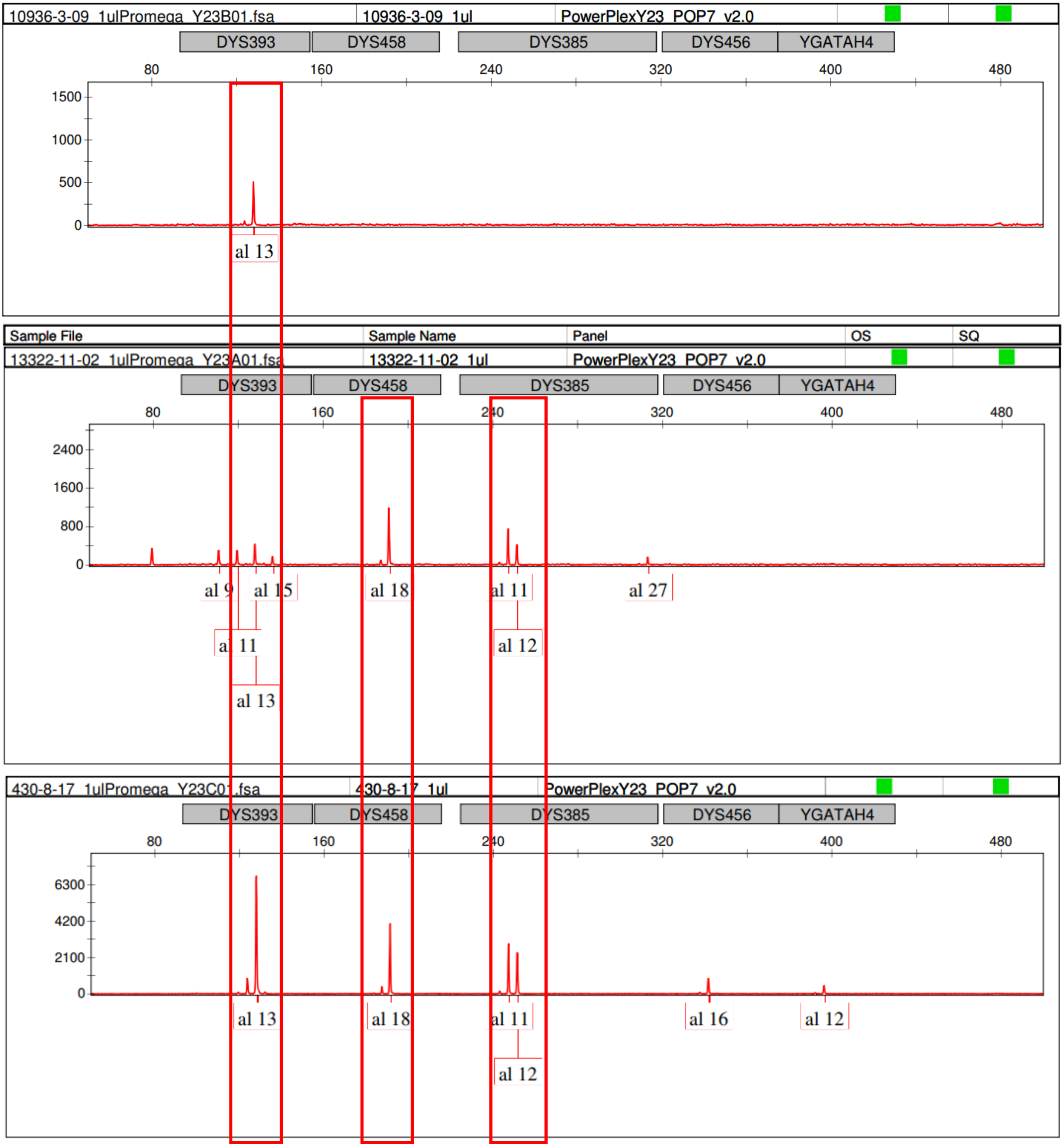
10936-3-09_1ulPromega_Y23B01.fsa
10936-3-09_1ul
PowerPlexY23_POP7_v2.0
DYS393
DYS458
DYS385
DYS456
YGATAH4
80
160
240
320
400
480
1500
1000
500
0
al 13
Sample File
Sample Name
Panel
OS
SQ
13322-11-02_1ulPromega_Y23A01.fsa
13322-11-02_1ul
PowerPlexY23_POP7_v2.0
DYS393
DYS458
DYS385
DYS456
YGATAH4
80
160
240
320
400
480
2400
1600
800
0
al 9
al 15
al 18
al 11
al 27
al 11
al 12
al 13
430-8-17_1ulPromega_Y23C01.fsa
430-8-17_1ul
PowerPlexY23_POP7_v2.0
DYS393
DYS458
DYS385
DYS456
YGATAH4
80
160
240
320
400
480
6300
4200
2100
0
al 13
al 18
al 11
al 16
al 12
al 12
